# Designing an App to Facilitate Self-Management in Young Adult Survivors of Childhood Cancer: Development and Usability Study

**DOI:** 10.2196/77121

**Published:** 2025-12-24

**Authors:** Meredith K Reffner Collins, Kristine Levonyan-Radloff, Jeffery McLaughlin, Margaret Masterson, Katie A Devine

**Affiliations:** 1 Division of Pediatric Hematology/Oncology Rutgers Cancer Institute Rutgers, The State University of New Jersey New Brunswick, NJ United States; 2 Radiant Creative Group Vienna, VA United States; 3 New Math Data LLC Houston, TX United States

**Keywords:** self-management, peer mentoring, cancer survivorship, long-term follow-up care, usability testing

## Abstract

**Background:**

Young adult survivors of childhood cancer are at risk for late and long-term effects from their treatment, and less than 1 in 5 obtain risk-based care in adulthood. Transitioning young adult survivors from pediatric, parent-driven care to adult, self-driven care is a challenging process during which young adults face multiple barriers. Intervening during this period may facilitate better transition readiness. For this purpose, we previously developed the Managing Your Health (MYH) web-based intervention, which showed initial feasibility and acceptability; however, young adult participants wanted to access the intervention through a mobile app.

**Objective:**

We used an iterative, cocreation design process to translate, build, and evaluate the usability of the MYH web-based intervention into a mobile app to be used in a future peer-mentoring educational intervention.

**Methods:**

In phase 1, we conducted key informant workshops with 3 stakeholder groups to understand target users’ needs and expectations related to the content and design of the mobile app. In phase 2, we conducted usability testing with young adult survivors of childhood cancer to evaluate the app’s usability and subjective appeal.

**Results:**

Participants in the key informant workshops (n=13) agreed that the content of the proposed app matched the barriers faced by young adult survivors of childhood cancer. Participants provided suggestions about the design of the app, including content and features, although there were mixed views about the inclusion of gamification features. Usability testing participants (n=25) rated the app highly on measures of technology acceptance, usability, and aesthetic appeal. Participants’ qualitative comments suggested that they found the app to be useful, easy to use, and likable or familiar relative to other existing apps. Participants suggested a variety of features to enhance the app, including adding features to enhance usability and reformatting certain aspects of the app to enhance interactivity and feedback to the user. Suggestions with uniformly positive reports were used to refine the app, while suggestions with mixed enthusiasm were not prioritized in refining the app.

**Conclusions:**

We engaged target users of an educational app in an iterative app design process to create a product that would meet the needs and expectations of those users. Results suggested that the app was generally viewed as acceptable, useful, and visually appealing. Common suggestions for improvement, such as reformatting quizzes to enhance interactivity and provide feedback regarding correct answers, were used to refine the app. The refined app will be used in the future intervention efficacy trial.

**Trial Registration:**

ClinicalTrials NCT06763770; https://clinicaltrials.gov/study/NCT06763770

## Introduction

Proper transition from parent-guided pediatric care to self-managed adult care is crucial for young adult survivors of childhood cancer. Nearly all cancer treatments can cause late effects that can occur months or even years after the treatment; these issues are specific to the type of treatment received [1,2]. Additionally, regardless of cancer type, childhood cancer survivors are at risk for greater morbidity and early mortality than their peers without a history of cancer. It is estimated that between 60% and 90% of childhood cancer survivors will develop at least 1 late effect, a quarter of which may be severe, disabling, life-threatening, or fatal [2-6]. To manage these late effects and to avoid additional complications, survivors of childhood cancers require lifelong survivorship care based on their treatment history and relevant risk factors. Unfortunately, less than 1 in 5 adult survivors of childhood cancer obtain risk-based care [7]. In fact, a recent survey found that only 27% of these individuals had a medical visit related to their cancer or treatment in the 2 years before the survey, and only 41% planned to complete such a visit in the subsequent 2 years [8].

The suboptimal usage of risk-based care is due, in part, to the challenges of transitioning to adulthood, a process that is demanding for all young adults (aged 18-29 years), regardless of cancer history. During this developmental period, young adults experience competing demands and milestones, such as leaving the childhood home, initiating a career, completing higher education, becoming financially independent, and starting a family [9,10]. However, in addition to these normal milestones, young adult survivors of childhood cancer must transition from pediatric-oriented care, which is typically managed by parents, to adult-oriented care, a setting in which the expectation is that the young adult survivors themselves manage their own health care.

There are many barriers to this transition from pediatric to adult care. Ideally, this transition would be treated as a *process* that occurs throughout adolescence. During this process, childhood cancer survivors would receive developmentally appropriate education regarding overall health management and lifestyle behaviors, as well as develop skills in communication, self-efficacy, medical self-management, and decision-making [11,12]. However, there are well-documented challenges in this process, which create poor transition readiness among young adult survivors of childhood cancer [3,13]; this suboptimal readiness can lead to inadequate follow-up or disengagement from care, which can exacerbate the impact of late effects on childhood cancer survivors’ lives [11].

To improve transition readiness among young adult survivors of childhood cancer, we developed a web-based self-management and peer-mentoring intervention [14]. Although young adult survivors of childhood cancer who participated in a randomized feasibility trial found the intervention to be feasible and acceptable, participants overwhelmingly preferred accessing intervention content through a mobile app [14]. These participants also wanted increased interactivity within the intervention modules, which could be accomplished through an app. Thus, we planned to develop the Managing Your Health (MYH) mobile app, to be deployed in a future iteration of the MYH intervention.

Digital health interventions, such as mobile apps, that promote, prevent, treat, and maintain health are a popular tool to use with young adults, aged 18-29 years [15]. Young adults are digital natives; almost 100% of this population uses the internet, with nearly 21% of this population accessing the internet using only a smartphone [16]. Due to the ubiquitous nature of digital tools in this population, meeting the needs of young adults who have been affected by cancer has become a popular target for digital health interventions [15,17,18].

Yet, *delivery* of digital resources does not automatically equate to *use* among this population. One of the best practices for developing digital health tools, such as mobile apps, involves soliciting input from or cocreating with the target audience of the app [19,20], especially among cancer survivors [14,17,18,21]. Working with potential users to create any digital health intervention increases the intervention’s personal relevance to the target user group and can better meet the community’s unique needs [17,22]. Additionally, potential users’ perceptions of the app’s usefulness, usability, appeal, and ease of use can be predictive of use among the target group [23-26]. As digital natives, young adults also expect well-designed, functional digital tools [27,28].

Therefore, we aimed to design a user-friendly app, “MYH 2.0” through 2 phases of work. The first phase used key informant workshops to inform the creation of the MYH 2.0 mobile app, and the second phase used usability testing to understand potential participants’ perceptions of the app’s design and usability.

## Methods

### Phase 1

#### Overview

To inform the app’s design and content, we used a 2-phased approach. In phase 1, we conducted focus-group style workshops with 3 groups of key informants and individual interviews with 2 consultants.

#### Recruitment

We included 3 key informant groups in the workshops, and recruitment methods varied based on participant type. Young adult survivors of childhood cancer were recruited through the mailing list of a long-term follow-up clinic at a National Cancer Institute–designated cancer center in the northeastern United States. Participants were eligible if they (1) had been diagnosed with any cancer between the ages of 0 and 21 years, (2) were between the ages of 18 and 29 years at the time of consent, (3) were treated in a pediatric cancer setting, (4) were at least 2 years from completing their cancer treatment, and (5) were able to speak and read English. Participants were excluded if they had a documented or self-reported cognitive delay that would prohibit completing the study or self-managing their health care.

Recruitment of health care providers and advocates occurred through the study team’s professional networks. To be eligible, participants had to be able to speak and read English.

#### Procedure

We conducted 3 key informant workshops, with participants assigned to a workshop based on their participant group. That is, young adult survivors of childhood cancer participated in one workshop, childhood cancer advocates participated in the second workshop, and health care professionals participated in the third workshop. The study team leader (KAD), a PhD-level researcher, and the app developer (JM) cofacilitated all workshops. Individual interviews were also conducted with an oncologist and a primary care provider who served as consultants to the research team. Due to the ongoing COVID-19 pandemic, all sessions took place via Zoom (Zoom Communications) in October 2021. These sessions were recorded, and they were, on average, an hour long.

The sessions focused on both generative (ie, brainstorming features, functions, and contexts of use) and reflective (ie, responding to preliminary designs) exercises and questions. Group moderators designed the sessions with guidance from the Liberating Structures framework [29]. Specifically, the moderators invited all participants to contribute, tried to balance participation levels among different participants, and tracked how time was allocated to different portions of the sessions [29].

#### Measures

Using an online survey, participants completed basic demographic measures following their participation in the workshops. Young adult survivors’ age, race, diagnosis, age at diagnosis, and time since diagnosis were all collected, while professionals and advocates only provided information about their professional roles.

#### Data Analysis

To analyze our key informant workshops, we relied on a text and topic summary approach [14,30]. This approach was selected because it matched the goal of the workshops: identifying strategies for turning the MYH website into an MYH app. Thus, we first transcribed each Zoom session. Then, members of the qualitative analysis team (KLR and 2 trained coders [Ivelisse Mandato and Melanie Spruill]) coded participants’ discussions into two primary categories: (1) reflections on the content of the existing program and (2) suggestions for improvement. Codes were then grouped to understand the overarching needs of the community relative to the app.

### Phase 2

#### Overview

In phase 2, we gathered feedback on the prototype of the MYH app by conducting individual usability testing interviews with young adult survivors of childhood cancer. These sessions were focused on the acceptance, usability, and subjective appeal of the app.

#### Recruitment

Eligibility criteria for young adult participants were the same as for the key informant workshops, although participants who had taken part in the key informant workshops were not eligible to participate in the interviews. All participants were recruited through the mailing list of a long-term follow-up clinic at a National Cancer Institute–designated cancer center in the northeastern United States.

#### Procedure

Before each session, participants received instructions to download the MYH app prototype, developed by Radiant Digital, to their personal phones, but were told that they would be provided with log-in information at the usability session. Both Android (Google) and iOS (Apple Inc) versions were available for download. Zoom sessions were conducted individually by 1 trained interviewer, an MS-level project manager on the study team (KLR). Interviews took place in May and June 2022. All sessions were recorded and were, on average, 47 (SD 5.74) minutes in length.

At the start of each session, study consent was reviewed, and participants gave oral agreement to participate. Then, participants were provided with a test username and password. Participants were asked to share their screen via Zoom so that the interviewer could follow along with their app usage. During the session, participants were asked to complete specific tasks within the app, such as logging in, editing a profile, locating certain features, etc, as well as to freely explore the app features. During the directed tasks and the free exploration, participants were instructed to “think aloud” throughout the session. After the session, participants completed a brief online survey with validated quantitative measures described below.

#### Measures

Participants completed the same demographic information collected from survivors in the key informant workshops, as well as the following measures.

#### Technology Acceptance Model

Participants’ perceptions of usefulness, ease of use, and behavioral intention were measured with items adapted from the technology acceptance model (TAM), which has previously been used to evaluate digital health tools among young adult cancer survivors [23,27]. Response options provided were a Likert-type scale, with 1 corresponding to *strongly disagree* and 5 corresponding to *strongly agree*. Higher scores corresponded to a more positive perception of the app feature.

Participants rated perceived usefulness with 5 items, including “The app will make managing my survivorship journey easier” (α=.76). Perceived ease of use was assessed with 4 items, including “I found it easy to get the app to do what I wanted it to do” (α=.78). Behavioral intentions were assessed with 3 items, including “I think that using the app in my own survivorship journey would be beneficial to me” (α=.80). Finally, use intentions were assessed with a single item: “I intend to use the app in my own survivorship journey.”

#### System Usability Scale

The System Usability Scale (SUS) is a validated measure that consists of 10 items, both positively and negatively worded, assessing the usability of the system [24-26]. The items were measured with a Likert-type scale, with 1 corresponding to *strongly disagree* and 5 corresponding to *strongly agree*.

To create the overall SUS score, the positively worded items were scored by subtracting 1 point from the participant’s response. The negatively worded items were calculated by subtracting the participant’s response from 5. The sum of the positive and negative items was then calculated and multiplied by 2.5 so that answers would range from 0 to 100. Scores are categorized as good (≥69), acceptable (51-68), or failing (≤50) [25,26].

#### Visual Aesthetics of Website Inventory

The Visual Aesthetics of Website Inventory – short form (VisAWI-S) was used to assess participants’ perceptions of the subjective appeal of the app [31]. The short form consists of 4 items, with each item representing a domain of the full VisAWI. All items were measured with a Likert-type scale, with 1 corresponding to *strongly disagree* and 7 corresponding to *strongly agree*. Higher scores represent a more positive impression of the aesthetics of the app (α=.76).

Specifically, participants assessed the simplicity, diversity, colorfulness, and craftsmanship of the app. Sample items included: “Everything goes together on this app” and “The layout appears professionally designed.”

#### Data Analysis

Qualitative data were analyzed using semantic, deductive thematic analysis [32-34]. Thematic analysis was selected for its iterative approach to the data. First, Zoom sessions were transcribed verbatim. Then, members of the analysis team (Ivelisse Mandato and Melanie Spruill) independently coded one assigned interview. Coders developed their own codes, and then the group met to compare the codes and code definitions developed individually. Audit trails documented these discussions. Each member recoded their assigned interview to finalize the codebook, and then the final codebook was shared with 2 main coders who independently coded each interview. After coding independently, these 2 coders met with the study manager (KLR) to resolve discrepancies using a consensus-building approach. All coding was done using Atlas.ti (Lumivero). All quantitative scale responses were analyzed using SPSS (version 29; IBM Corp). Descriptive statistics (eg, mean and SD) were calculated. We present the quantitative data with the qualitative data with which it best aligns in the Results section of this study.

### Ethical Considerations

The Institutional Review Board at Rutgers University approved this study before launch (IRB #Pro2021001514). Participants provided informed consent before their participation. Identifiable information for all participants was stored on the University’s enterprise Health Insurance Portability and Accountability Act–compliant cloud system (ie, Microsoft OneDrive), and only authorized team members had access to the information. Once the data analysis was completed, all individually identifiable information was destroyed; the data presented here are deidentified. Each participant was compensated with a US $50 electronic gift card as a thank you for their time.

## Results

### Phase 1

#### Overview

We engaged a total of 13 participants, including 7 young adult survivors of childhood cancer, 3 advocates, and 3 health care providers involved in the care of childhood cancer survivors. Young adult survivors were between 23 and 26 years, and they had been, on average, 9 (SD 6.3, range 0-17) years old at diagnosis. There were 2 female and 5 male participants, 3 (43%) of whom identified as racial minorities. In total, 6 of the 7 (85.7%) young adult survivors experienced blood cancers. Time since diagnosis was 15.6 (SD 5.7) years.

The 3 advocates included a patient advocate, a parent advocate, and a nonprofit advocacy organization leader. The 3 health care providers represented the professions of oncology, social work, and psychology. We also individually interviewed 2 consultants on the project (representing leaders in oncology and primary care).

#### Theme 1: Content Matches Current Barriers

Overall, our key informants found that the content of the MYH program matched the barriers that were salient during the transition period. Both young adult survivors and the health care providers endorsed this theme. Participants found that the content addressed behavioral and psychological (ie, low self-efficacy and feeling “different” from healthy peers), knowledge (ie, the transfer of medical knowledge from parent to adult), and structural (ie, disjointed communication with providers, lack of primary care providers with training in survivorship, and health insurance) challenges.

The advocates suggested adding content relevant to parents and primary care providers. They also suggested adding content that was more tangential to the transition. Given that data collection took place during the COVID-19 pandemic, advocates also suggested including resources specifically for survivors related to staying safe during the pandemic.

#### Theme 2: Design and Feature Recommendations

All participants, regardless of informant type, rated the subjective appeal of 2 user interface design styles via an anonymous poll during their key informant session. All participants—survivors, providers, and advocates—reported liking the general appearance of both styles, although there was a strong consensus among survivors toward one of the two styles, which was selected for further development.

All participants also offered recommendations for the design and features of the proposed app. Participants recommended designing the app with a simple, easy-to-use interface, offering a checklist of tasks to complete, a glossary of medical jargon, and tools for educating and sharing information with primary care providers. Participants were also highly interested in seeing tools to help navigate and understand health insurance included in the app, and they also wanted the app to include tools for managing stress.

Health care providers also noted that the needs of young adult survivors of childhood cancers change over time as individuals’ health status also changes. For example, fertility may become an important concern when young adult survivors are ready to start a family. Needs may also change as late effects begin or worsen. Health care providers, then, wanted to add “nudges” to remind survivors to return to the app at different points in their lives, especially when certain material becomes more relevant to their life stage (eg, at the time of starting a family).

Participants of all key informant groups reported mixed reactions to gamifying the app. Some participants felt that gamification was a helpful feature that could motivate potential users to complete the program, while others felt that adding gamification elements seemed too trivial for the subject matter. In other words, those participants felt that gamification made light of the serious nature of cancer survivorship care. Among potential gamification features, offering completion badges in exchange for finishing content modules was the lowest-rated feature presented.

#### Data Synthesis: Development of the MYH App Prototype

Using the findings from the key informant workshops, we developed the MYH app. Since informants agreed that the MYH web content was still relevant and applicable to the barriers faced during the transition period and that the MYH web-based intervention was found to be engaging and acceptable, the *content* of the app was largely copied from the existing web-based intervention [14,35]. We retained the 5-module structure of the original, web-based MYH program, with minor content updates to reflect the most accurate health care guidance. Table 1 describes the 5 modules and their associated learning objectives; development of this content is described in the study by Viola et al [14].

**Table 1:** Modules and learning objectives of the Managing Your Health program.

Module	Module name	Module learning objectives
1	Understanding your survivorship care plan	Name your diagnosis, treatments received, and risks for late health effects. Obtain (if needed) and store your survivorship care plan. Identify any health screenings you need going forward and how often you need them.
2	Taking charge (of your healthcare)	Identify your strengths and weaknesses regarding self-management tasks. Discuss the value of establishing and maintaining a relationship with your primary care provider. Explain your health insurance coverage. Identify strategies for overcoming any barriers to obtaining care.
3	Thoughts and feelings	Discuss strategies for coping with uncertainty. Name the 5 steps of problem-solving. Identify your own feelings about being a survivor and requiring lifelong care. List effective strategies for dealing with stress and managing your time.
4	Getting support	List the ways family involvement in your care is helpful. Identify specific tasks for which you would like to assume more responsibility. Discuss strategies for negotiating responsibility for care with your family. List effective communication strategies.
5	Staying healthy	List recommendations for physical activity and determine if you are getting enough exercise. Describe healthy nutrition and sleep habits. Identify the benefits of avoiding risky alcohol and substance use. Discuss safe sexual health practices. Name ways to stay sun safe and protect your skin.

The Radiant Digital team built the app’s architecture using STRAPI, an open-source, node-based content management solution. Because no protected health information was to be collected during the individual usability interviews, the parallel architecture for processing and retaining protected health information was not integrated into the app at this stage.

Based on the key informant workshops, we created a list of design and feature priorities. We used the survivor-informants’ preferred design style as the base of the app’s aesthetic design. During this phase, we prioritized the development of features that had clear, uniform support from our key informants. For example, because our key informants were ambivalent about gamification during the workshops, we did not include this feature.

**Figure 1:**
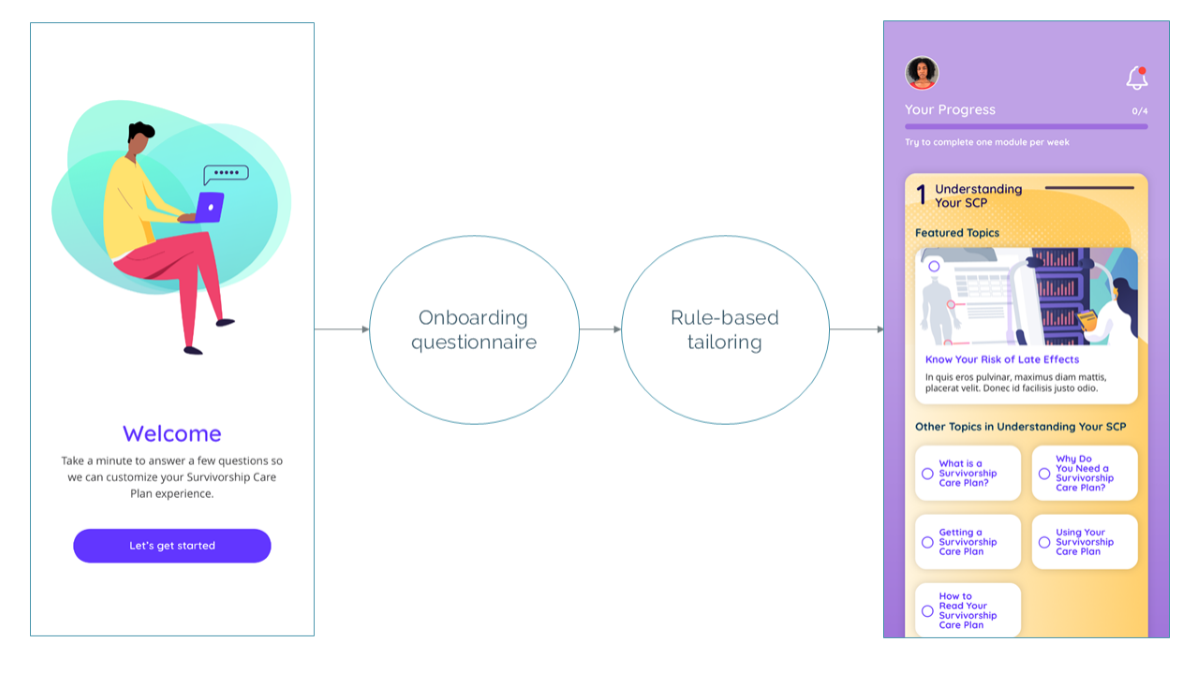
Rule-based tailoring was used to highlight relevant subtopics based on user responses to the onboarding questionnaire.

Our key informants uniformly expressed a desire for content to be tailored to their personal needs and experiences. To highlight content of high relevance to each user, we created an onboarding survey to welcome users to the app and assess their current needs or interests across the topics covered in the modules. Responses to the onboarding survey were used to reorder content and highlight key, personally relevant subtopics within each module. Users retained access to all content, but certain subtopics were highlighted for review based on responses. For example, the onboarding survey asks users about their confidence in following their survivorship care plan. Reporting low confidence would highlight the relevant subtopic. Figure 1 illustrates how the onboarding questionnaire used rule-based tailoring to highlight key subtopics within each module.

Our key informants also uniformly expressed a desire for interactive features. To accomplish this, we added “quizzes” and “Just Checking In” surveys within each content module that focused on evaluating knowledge of the content presented or encouraging self-reflection on how the content applies to their own lives. Where knowledge acquisition was the focus, such as with learning health insurance terms, “quizzes” were used to test knowledge and provide corrective feedback as needed. Where self-reflection was the focus, “Just Checking In” surveys used a combination of questions with multiple response options and open-ended responses to encourage self-reflection. For example, module 3 discusses coping strategies, and the “Just Checking In” survey asks users to check the coping strategies that work well for them and select a coping strategy to practice.

**Figure 2:**
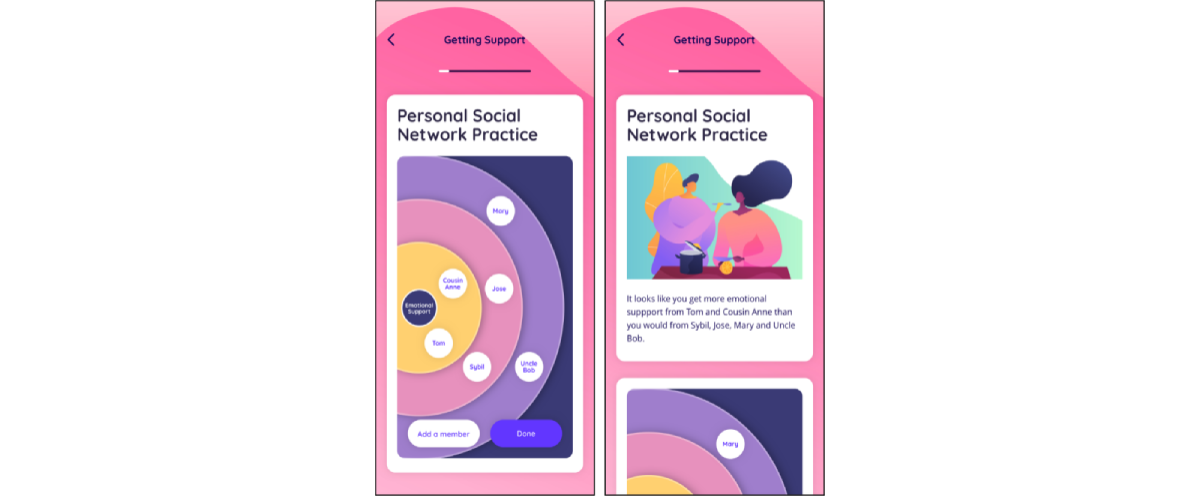
Interactive “Personal Social Network” exercise within the Managing Your Health app.

We also added an interactive activity aligned with one or more learning objectives to each module. For example, in module 4: *Getting Support*, we added a personalized social network builder that allows young adult survivors to visualize who in their social network offers support along various support domains (eg, emotional, practical, and informational). The tool allows participants to place support members in an inner, middle, or outer ring that corresponds to frequency and effectiveness of support interactions with the named social network member as a way to actively reflect on their social support, instead of reading text about it (refer to Figure 2 for screenshots of the activity).

We also recognized the importance of privacy and confidentiality within the app. We added profile management features to ensure that young adult survivors felt in control of their information and personal goals. Users decide what information they want to put into the app, and notifications alert users to re-engage with the app without providing any specific, identifiable user information.

**Figure 3:**
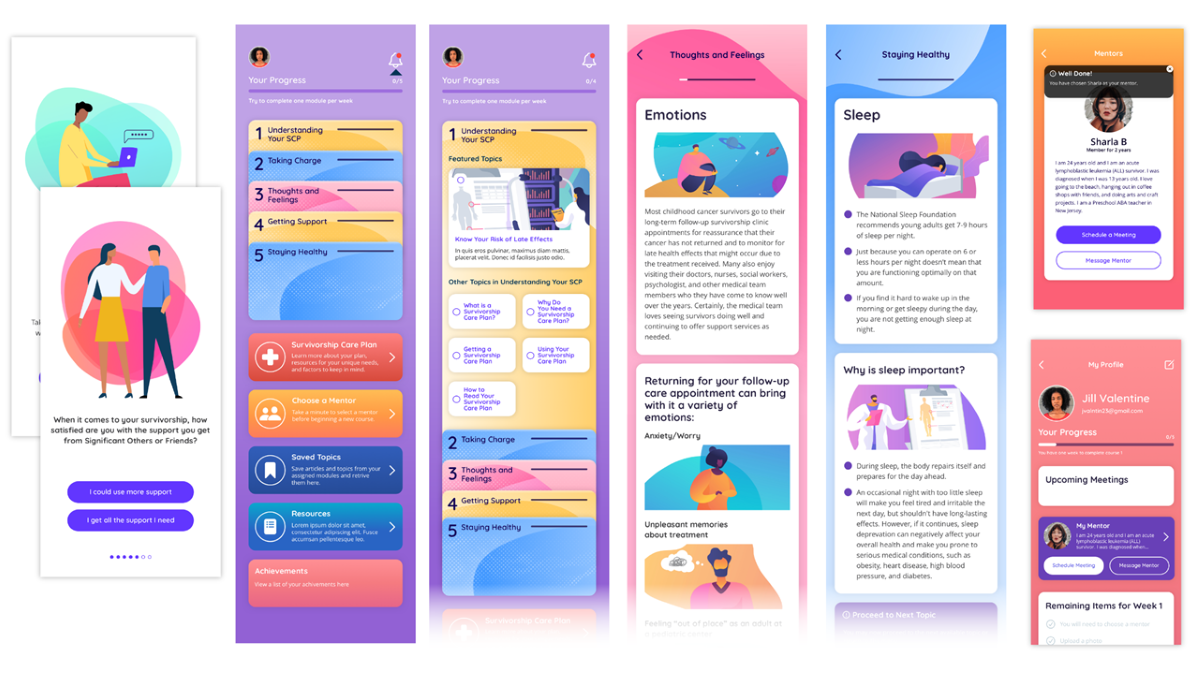
Screenshots demonstrating the onboarding survey, dashboard layout, sample module content, and mentor features of the Managing Your Health app prototype.

In-app progress tracking also had uniform support among our key informants, so we added a progress tracker so that young adult survivors could see and understand how each module fit within the whole program. An overview of the overall design and content of the app is illustrated in Figure 3.

### Phase 2

#### Overview

Interview participants (n=25; Table 2) were young adult survivors of childhood cancer. Participants, on average, were in their early to mid-20s (mean age 24.08, SD 1.91 years), and they were between 5 and 24 years since diagnosis (mean 13.56, SD 5.51 years). Participants were primarily diagnosed with blood cancers (n=18, 72%). More than half identified as male (n=15, 60%), and a little more than three-quarters of participants identified as Hispanic White (n=3, 12%) and Non-Hispanic White (n=16, 64%).

**Table 2:** Usability interviews participant demographics (n=25).

Demographics		Value
Age, mean (SD)		24.08 (1.91)
Age at diagnosis, mean (SD)		10.45 (5.44)
Years since diagnosis, mean (SD)		13.56 (5.51)
**Sex, n (%)**		
	Male	15 (60)
	Female	10 (40)
**Ethnicity, n (%)**		
	Hispanic	3 (12)
	Non-Hispanic	22 (88)
**Race, n (%)**		
	Hispanic White	3 (12)
	Non-Hispanic White	16 (64)
	Non-Hispanic Black	2 (8)
	Non-Hispanic Asian	3 (12)
	Non-Hispanic more than one race	1 (4)
**Cancer site**		
	Blood	18 (72)
	Central nervous system	4 (16)
	Neuroblastoma	1 (4)
	Sarcoma	2 (8)

Most user feedback was positive, indicating high acceptance and high perceived usefulness of the app. Three primary themes that were identified were (1) useful and easy, (2) likeable and familiar, and (3) suggested changes and improvements.

#### Theme 1: Useful and Easy

Participant interviews highlighted the usefulness of the *content* and *features* of the app (refer to Table 2 for an overview of the themes with additional exemplar quotes). They found the module that would allow participants to create a step-by-step, personalized action plan to be particularly helpful. Two of the most liked features explained how to make an appointment with a primary care physician and how to navigate health insurance; these features also came with interactive components with which users could practice by applying their knowledge. For example, participants commented on the utility of the app, providing a sample script to follow in making a health care appointment. One participant said:

I like the fact that you’re able to print out the form [script]. It kind of tells you what to say and then you can just print it out. It’s cool! Normally I get nervous trying to like make sure I say everything perfectly. I’m so used to my mom making appointments for me.

Non-Hispanic Black female, 20 years, leukemia

Participants also described how the in-app insurance term glossary and example labeled insurance card were helpful. One participant remarked:

This is probably the most confusing part of being on your own medical plan. This is a nice summarization; I feel like it can be overwhelming for new plan users. It can get all confusing about premiums, deductibles, copays – all that stuff – these are definitely the toughest topics.

Non-Hispanic White male, 26 years, neuroblastoma

Participants also found the content of the app *easy to digest*, which likely contributed to perceptions of the app’s usefulness. Participants noted that the personable presentation of the content was positive, and the use of videos and images to supplement and further explain written information was also noteworthy. For example, one participant said:

I like how you guys have videos cause sometimes I don’t know what I have to do. Reading it is one thing, also seeing it is much better.

Non-Hispanic White female, 26 years, leukemia

Participants’ quantitative results reflected their overall comments about the app (Table 3), which was perceived as useful (mean 4.49, SD 0.40). Participants thought that using the app would be beneficial to them (mean 4.55, SD 0.45), and they reported strong intentions to use the app if available to them (mean 4.44, SD 0.65).

**Table 3:** Usability interviews illustrative quotes by theme.

Theme	Definition	Exemplar quotes
Useful and easy	The *content* and *features* of the app were perceived as useful to managing survivorship and easy to use	“I thought that that was particularly very good, kind of like a little breakdown of how you could talk to your doctor about it, because it’s not easy doing it for the first time when you’re on your own. So especially after you had a mom or dad helping you out for most of your life” [Hispanic White male, 23 y, lymphoma] “I thought it was a good amount of information and broke down what we need to know, while taking those steps to take [sic] care of your health yourself. Especially with the insurance information and then breaking down hot to get a doctor’s appointment, what to do, what to say. ‘Cause I feel like that is probably anxiety producing for people who haven’t done it before” [Non-Hispanic White female, 25 y, leukemia] “And yeah, just understanding these what these simple terms mean, because I feel like, like for example, like coinsurance, copay, like I feel like, sounds, similar people could get confused, so having this laid out like a little glossary. Very helpful and then I like the card explanation. I'd like your medical card. I feel like sometimes when I fill out information like I'm going to like a new Doctor for the first time or whatever. I always have to make sure. Oh that's my like member ID number whatever. And this kind of clearly explains what it what, so that's helpful” [Non-Hispanic Asian male, 21 y, leukemia] “I like that it really, really emphasizes the ones that were relevant to me” [Non-Hispanic Black male, 25 y, leukemia] “It makes it easy to kinda identify the important key points, and if it is too long, there is a good chance I just wouldn’t read it. and so I liked how it kinda broke everything up into a little sections” [Non-Hispanic Asian male, 25 y, lymphoma] “I think it’s really- I like how it’s designed and like how you’re not seeing too much all at once. Like you’re going page by page with the modules that feels very like digestual with the information” [Non-Hispanic White female, 25 y, leukemia] “Anything that’s like short and sweet information like this is nice … even though it’s multiple paragraphs - I’d rather have a short decent paragraph rather than a long. Because I’ve googled stuff and then you get a three page paper and I’m like never mind” [White male, 23 y, leukemia]
Likeable and familiar	The *design* and *aesthetics* of the app were perceived as likeable and familiar to potential users	“This kind of reminds me too – I have a meditation app – and this is kind of how they list all their different options of meditation classes you can take, which I think is why I was just clicking” [Non-Hispanic White female, 22 y, brain tumor] “It was good, it’s very well laid-out, it’s very clear. It reminds me of Duolingo, which is pretty good” [Non-Hispanic White male, 27 y, lymphoma] “So overall, really easy to use, accessible and I feel like definitely a lot of people will find this helpful and useful” [Non-Hispanic Asian male, 21 y, leukemia] “I thought it was good and was very user friendly, was easy to use. No, I like it, I like how vibrant and user friendly and engaging it is” [Non-Hispanic White female, 24 y, lymphoma] “Based on my hour using this, I can’t think of a better design myself. It’s simple – maybe some people like more complicated looking apps but I like how it’s simple and easy” [Non-Hispanic White male, 26 y, neuroblastoma] “I think it’s super easy to use, I am very comfortable using it, very comfortable navigating. I don’t think it’s any more difficult than any app that you’re going to be using on the entire market” [Non-Hispanic White male, 23 y, leukemia]
Suggestions and improvements	Participants suggested both *content* and *layout* additions to increase the app’s relevance to survivorship care	“The last portion when it’s asking you about the things you don’t like about the provider – what is the point of that? I feel like it didn’t give you any feedback. You know it’s just asking you how comfortable you are with your provider and then it’s just like- kind of leaves it there” [Non-Hispanic White male, 26 y, lymphoma] “Since I just mentioned that all that information is kind of sensitive to keep on the phone, I was thinking ...this might be kind of tedious for people, I was think if you could like...log in to see just that part, or something, login like a hospital, I don’t know....” [Non-Hispanic White male, 22 y, brain tumor] “When you say like type, rather than type may it would just come up with a calendar, right? That would probably be helpful, especially if you can sync it to your phone calendar, although that might be kind of a big ask. I don’t really know if that’s easy to do or not, or if at least just, you know, pull up the date right?” [Non-Hispanic Asian female, 26 y, leukemia] “For this app, I would turn on push notifications where it would go on my phone and say ‘you still have things to complete for Week 1. Complete them so you can move onto Week 2’” [Non-Hispanic White female, 22 y, brain tumor] “Yeah, I do like the bullet points… I feel like there’s- you could add more [info] for symptoms for primary care” [Non-Hispanic White female, 25 y, leukemia] “Ok when I choose the wrong answer, it still looks the same as to if I were to choose the right answer. I feel as if I chose the wrong answer, it will be really helpful to maybe have a text box at the bottom saying “incorrect”- just saying like “incorrect” and giving the definition to what I chose that was incorrect. And the correct answer to have definition again, just so I kind of reread it” [Non-Hispanic Black female, 20 y, leukemia] “I would want to I guess explain more on why that’s the answer. And like why it’s not the answer I chose” [Hispanic White female, 23 y, sarcoma] “Because it’s the same color or font as the number of two years, so it feels like they’re equally as important, but I feel like the name is more important than how long they’ve been a member. So maybe italicize member for two years and bold the name” [Non-Hispanic Black male, 25 y, leukemia]

Overall, participants found the app to be applicable and useful to their survivorship experience, providing them with interactive resources that they could use again, increased the applicability of the app’s content and features. Table 4 shows the ratings of the TAM among usability interview participants.

**Table 4:** Technology acceptance model ratings among usability interview participants (n=25).

Subscale and item^a^			Mean (SD)
**Perceived usefulness** (α=.76)			4.49 (0.40)
	The app will be useful to help me manage my survivorship journey	4.64 (0.49)	
	The app will make managing my survivorship journey easier	4.64 (0.49)	
	The app will help me to understand the survivorship topics I'm interested in	4.44 (0.58)	
	The app will help me in my interactions with doctors, insurers and others	4.28 (0.68)	
	The app will help me to manage my own survivorship self-care	4.44 (0.58)	
**Perceived ease of use** (α=.78)			4.53 (0.43)
	The process of learning to use the app was easy for me	4.72 (0.46)	
	I found it easy to get the app to do what I wanted it to do	4.40 (0.65)	
	I found the app to be clear and understandable	4.44 (0.58)	
	I found the app to be easy to use	4.56 (0.51)	
**Behavioral intentions** (α=.80)			4.55 (0.45)
	I think that using the app in my own survivorship journey is a good idea	4.52 (0.51)	
	I think that using the app in my own survivorship journey would be beneficial to me	4.56 (0.58)	
	I have a positive impression about using the app	4.56 (0.51)	
**Use intentions**			
	I intend to use the app in my own survivorship journey	4.44 (0.65)	

^a^All items were measured with the following: 1=*strongly disagree* through 5=*strongly agree*. Higher scores meant more positive perceptions of the feature.

#### Theme 2: Likeable and Familiar

Participants were highly satisfied with the design of the app—the layout and the aesthetics. They felt the design was *likeable and familiar*, and there was a clear reciprocal relationship between the 2 components of this theme; that is, the likeability and familiarity of the app seemed to contribute to and reinforce each other in participants’ minds.

Participants liked that the app was well-organized, accessible, and user-friendly. They perceived that the layout and navigation particularly exemplified these adjectives. For example, one participant said:

The flow of the app is actually one of the things I really like the most. I think it just works very well, in a lot of the different ways. A lot of different pages will segue into each other kind of seamlessly … I don’t think anyone will have any issues using it. It’s very well laid out.

Hispanic White male, 23 years, lymphoma

A second participant described the layout:

I love the layout, how it’s just kind of … It’s organized.

Non-Hispanic Black female, 20 years, leukemia

A third participant discussed how it was beneficial to have multiple touch points for the same information:

I like things being in two places. So, if I misremember … [the information] being in a very similar format so it makes it kind of a catch.

Non-Hispanic Black male, 25 years, leukemia

In other words, participants appreciated how the app was designed and how it functioned.

Additionally, several participants compared the design of this app to other apps widely available in the app store, such as the language-learning app Duolingo. These design similarities created a sense of familiarity among our participants; that is, the overall aesthetic and navigational design of our MYH app reminded participants of other mass-market apps. Participants’ sense of familiarity with the app seemed to increase the likability of the MYH app, as participants felt more confident in their ability to navigate the app and take full advantage of its features—all key components of the SUS measure. For example, one participant said:

It was pretty easy – when you [the interviewer] asked me to do something I felt like I knew pretty roughly. Like I know that little box with the pencil is an edit button so if you’re on your profile I knew it would be to edit my profile.

Non-Hispanic White male, 26 years, neuroblastoma

Thus, one important component of participants’ positivity toward the app was a design that was like other mass-market apps.

**Figure 4:**
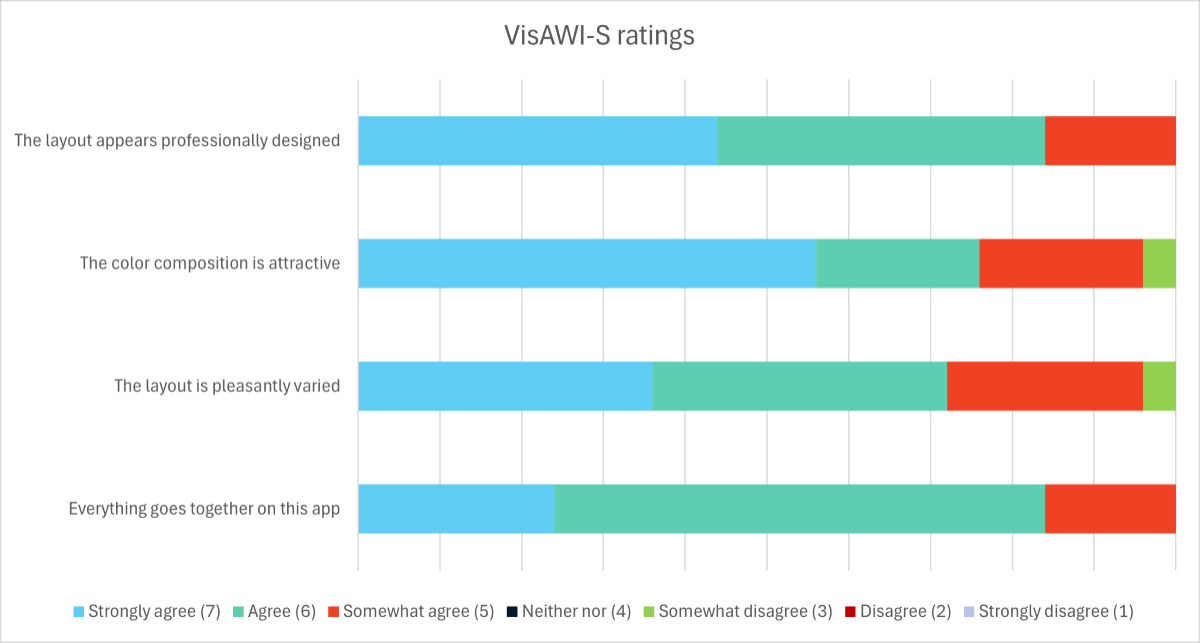
Participants’ perceptions of the subjective appeal of the MYH app. VisAWI-S: Visual Aesthetics of Website Inventory – short form measured on a 1 (strongly disagree) through 7 (strongly agree) scale.

**Figure 5:**
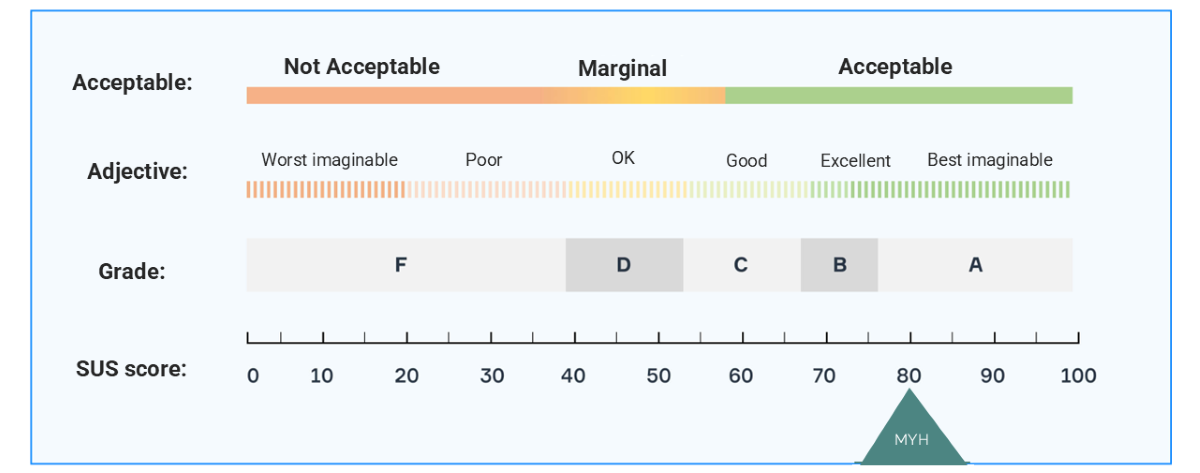
Participants’ ratings of the Managing Your Health app on the System Usability Scale (range 0-100). MYH: Managing Your Health; SUS: System Usability Scale.

Again, participants’ quantitative results reflected their qualitative comments. The app was perceived as visually appealing (mean 6.15; SD 0.86) and easy to use (mean 4.53, SD 0.43; Figure 4). Participants’ overall usability rating on the SUS was 80.3 out of 100 (SD 11.55), which can be categorized as “Excellent” (Figure 5).

#### Theme 3: Suggested Changes and Improvements

Finally, participants described suggested changes and improvements that they wanted to see in the app. This theme referred to *content* (eg, unnecessary features and desired additions) as well as *layout* (eg, formatting changes).

Participants’ suggestions addressed a range of functionalities that they thought would make the app both more useful and easier to use. For example, multiple participants wanted to integrate a calendar feature within the app. For example, one participant said:

Maybe there should be like a calendar function where you can, you know when your appointments are, you can put them on this one app and you are not juggling between your calendar app, or email, it would kinda be centralized in here [the app].

Non-Hispanic Asian male, 25 years, lymphoma

A second participant (Non-Hispanic White female, 26 years, medulloblastoma) suggested integrating a medication list within the app so that survivors could easily report which medications they were taking at appointments because it would be “just an easier way to help them [other survivors] manage their medicines,” while a third participant (Non-Hispanic White female, 26 years, leukemia) wanted a to-do list integrated in the app so user could notate “what you need to ask your doctor or what symptoms you are having.” These features were suggested as ways to consolidate the information that our participants felt they needed to have on hand—often from their own experience of follow-up care—but the app did not currently include.

Participants also suggested changes to the *formatting* of the app’s content. This feedback varied, although multiple participants requested reformatting the quizzes throughout the app’s modules. For example, one participant said:

Yeah, I probably prefer like if I were to get the answer wrong, it would like highlight it in like a red color. Just show me that it’s wrong and then I could click on something and then like and something pops up and it just kind of gives an explanation on why your answer was wrong and then like what’s the correct answer.

Non-Hispanic Asian male, 21 years, leukemia

Participants wanted to understand *why* they missed quiz questions, further indicating the usefulness of the app’s content. They asked for incorrect answers to be more easily identifiable, and they wanted explanations. To our participants, these formatting changes would help them learn—and internalize—the content so that they could use it in their lives.

Other formatting suggestions included making the app more inclusive. One participant suggested adding,

…some sort of gender-neutral sign in the middle … because it’s like ‘man and woman’ but that’s not very inclusive of members of the trans[gender] community or [the] non-binary community.

Non-Hispanic White female, 24 years, lymphoma

Overall, participants gave both positive quantitative and qualitative feedback about the content and design of the app; this feedback suggested high usefulness and ease of use among this population.

## Discussion

### Overview

In this study, we developed and evaluated the content and usability of the MYH app. The app is one component of a proposed transition-readiness intervention for young adult survivors of childhood cancer. The purpose of the intervention is to promote effective transition from parent-guided pediatric survivorship care to self-guided adult survivorship care through interactive, personalized educational content within the app and a peer mentor to connect with on videoconference outside of the app. Overall, our participants quantitatively and qualitatively reported that the MYH app contained content that was relevant and useful to their experience as young adult survivors of childhood cancer and was visually appealing and easy to use.

### Principal Findings and Comparison With Previous Work

We implemented an iterative design process to ensure that app development met the needs and expectations of our target users, young adult survivors of childhood cancer. Engaging target users early in the design process, especially for the purpose of understanding their specific needs and wants, is essential for the implementation and uptake of mobile health technologies [36,37]. Additionally, in a previous study with young adult survivors of childhood cancer, the acceptability of a survivorship health management app was positively and moderately correlated with app engagement [38].

Participants in the phase 1 key informant workshops affirmed that the content in the MYH program matched the barriers faced by young adult survivors of childhood cancer. Participants were also highly satisfied with the preliminary designs for the app, especially the proposed features intended to help navigate the complexities of health insurance coverage. Health insurance is consistently a source of stress for young adults, regardless of cancer history, and studies have demonstrated that people in this age group have low knowledge and self-efficacy [39,40]. Childhood cancer survivors are also more likely to experience financial hardships [41,42]. The insurance navigation feature is just one example of how the unique needs of young adult survivors of childhood cancer were met by the content of the app.

Participants provided mixed feedback about gamifying the app, which came as a surprise given previous literature suggesting that gamification can increase engagement and goal achievement in cancer survivors [21,43,44]. Additionally, gamification elements have been positively associated with intrinsic need satisfaction, such as autonomy competence, motivation, and relatedness [45]. However, there is a lack of clear efficacy data in relation to gamification for disease self-management among this population; among interventional apps that include gamified elements, efficacy studies are rarely designed or adequately powered to understand the effects of gamification on study outcomes, leaving the question of whether it is the gamified design or another element driving efficacy [46-48]. Since increasing engagement is often stated as the primary rationale for including gamified elements among other eHealth tools—and the tailored, interactive features of the MYH app could spur such engagement—we decided to develop the app without any additional gamification elements, such as point rankings, completion badges, and so on [48].

Usability testing sessions with 25 young adult survivors of childhood cancer established high acceptance and perceived the usefulness of the app. Participants’ qualitative feedback during the sessions related to three categories: (1) useful and easy, (2) likeable and familiar, and (3) suggested changes and improvements.

Overall, participants’ qualitative comments indicated that they found the content of the app to be useful in their survivorship journey and easy to understand. Participants also found the design and navigation of the app to be likeable and familiar (ie, similar to the design of other apps they already use). These qualitative remarks aligned well with quantitative scores across the TAM and VisAWI-S measures, which indicated that most participants agreed or strongly agreed that they perceived the app as visually appealing, useful, and easy to use. High ease of use was also evident from participants’ ratings on the SUS, which was higher than the industry average of 68 across a wide range of apps [49,50]. Since perceived usefulness is a key driver of technology use, especially among this population, this feedback is encouraging for future use of the MYH app [17,23].

Our participants provided the most enthusiastic feedback about features that could help them manage their survivorship care; they also proposed additional app features based on their real-life experience of accessing survivorship care. Because these comments primarily focused on enhancements to the existing app features rather than limitations or irrelevant features, it seems that participants already saw the MYH app as a feasible and usable tool through which to manage their survivorship care. Additionally, young people can be overwhelmed by the sheer amount of information available online and want to be able to quickly find information that is relevant to their unique situation [51]. As demonstrated by their responses, participants in our study were most drawn to features that they would use and were most likely to suggest features that they wished they had available to them earlier in their transition process. Taken together, these findings echo previous research emphasizing the importance of personalized information and resources within digital health tools [19,20], especially for cancer survivors [14,17,18,21].

### Limitations

This study, like all studies, is not without limitations. First, despite our attempts to recruit a diverse group of participants, most participants in both the key informant workshops and the usability interviews predominantly identified as Non-Hispanic White, which may indicate that our findings do not reflect the opinions of all young adult survivors of childhood cancer. Second, we recruited our sample primarily from one National Cancer Institute–designated cancer center in the northeastern United States, although our advocates in the key informant workshops were from various parts of the country. This may also limit the applicability of our findings to all young adult survivors of childhood cancer, especially those who come from other parts of the country. Third, to move quickly through the app development, our key informant workshops relied on a text and topic summary approach to data analysis, which may have obscured additional nuances within the data. Finally, data collection occurred during the COVID-19 pandemic (ie, October 2021-June 2022), which may have altered participants’ views on salient features and needs in unknown ways.

### Future Directions

Based on the feedback provided in the individual usability interviews, the team designed and implemented several changes to the functionality, layout, and content of the app. The team also added both backend architecture and functionality to manage any potential security concerns, including a PIN sign-in and industry-standard security.

Because previous testing established the high feasibility and acceptability of the MYH program (ie, the intervention modules and the peer mentor), conducting a randomized controlled efficacy trial of the program is a critical next step in contextualizing and translating the results presented here [35]. Due to the relative homogeneity of the sample in the design phase, as presented here, recruiting from multiple sites across the country was prioritized in the design of the efficacy trial, which is currently underway.

### Conclusions

We engaged young adult survivors of childhood cancer in an iterative cocreation process to ensure that the MYH app reflected the needs and expectations of the target population, as is best practice in developing mobile health technologies for specific populations [17,22,36,37]. The key informant workshops and usability testing demonstrated that the MYH app is useful, visually appealing, and easy to use among young adult survivors. While participants rated the proposed design and features highly, they also had many—and varied—suggestions for content and layout improvements that would enhance the existing app. These results were used to refine the app for further efficacy testing.

## Data Availability

The deidentified data analyzed during this study are available from the corresponding author on reasonable request.

## Abbreviations

MYH: Managing Your Health
SUS: System Usability Scale
TAM: technology acceptance model
VisAWI-S: Visual Aesthetics of Website Inventory – short form
